# Characterisation, Chain Conformation and Antifatigue Effect of Steamed Ginseng Polysaccharides With Different Molecular Weight

**DOI:** 10.3389/fphar.2021.712836

**Published:** 2021-07-27

**Authors:** Lili Jiao, Junming Li, Furao Liu, Jing Wang, Peng Jiang, Bo Li, Hui Li, Changbao Chen, Wei Wu

**Affiliations:** ^1^ Jilin Ginseng Academy, Changchun University of Chinese Medicine, Changchun, China; ^2^ The Affiliated Hospital Changchun University of Chinese Medicine, Changchun, China; ^3^ National Demonstration Center for Experimental Biology Education, Northeast Normal University, Changchun, China; ^4^ College of Pharmacy, Changchun University of Chinese Medicine, Changchun, China

**Keywords:** steamed ginseng, polysaccharides, solution conformation, antifatigue activities, exercise-induced fatigue

## Abstract

Two polysaccharides were obtained from steamed ginseng *via* ultrafiltration, and their physical–chemical properties, solution properties and antifatigue activities were studied. WSGP-S3 and WSGP-G3 were acid heteropolysaccharides with the molecular weights of 2.03 × 10^4^ and 4.86 × 10^4^, respectively. They were composed of different molar ratios of the monosaccharides Rha, GlcA, GalA, Glc, Gal, and Ara. The results of size-exclusion chromatography–multiangle laser light scattering analysis, Conge red staining and Circular dichroism spectroscopy revealed that WSGP-S3 exhibited a random conformation of branched clusters in solution. By contrast, WSGP-G3 exhibited an ordered conformation, including helix-like conformations in aqueous solution. Antifatigue activity tests proved that WSGP-S3 markedly prolonged the exhaustive swimming time of fatigued mice; increased liver and muscle glycogen levels and superoxide dismutase, catalase, glutathione peroxidase activities and decreased blood lactic acid, nitrogen and malondialdehyde levels compared with the control treatment. Moreover, it enhanced spleen cell proliferation in fatigued mice. By contrast, WSGP-G3 had no significant effect on fatigued mice. The results showed that WSGP-S3 might have a major contribution to the antifatigue effects of steamed ginseng polysaccharides and could be a potential anti-fatigue polysaccharide.

## Introduction

Fatigue is a complex physiological phenomenon that is an important factor affecting bodily functions. It is caused by many reasons, including the excessive consumption of energy, the production and accumulation of energy metabolites, the disorders of the immune system and oxidative damage (Azizbeigi et al., 2014; Chi et al., 2015). Fatigue can cause the disorders of the nervous system; reduce learning and work efficiency and seriously affect emotional, physical and mental health. In addition, it can cause a variety of complications, such as aging, cancer, depression and Parkinson's disease (Tharakan et al., 2006). Therefore, the search for natural antifatigue agents with nontoxic side effects to eliminate fatigue has attracted increasing attention.

*Panax ginseng* C.A. Meyer belongs to Araliaceae. It has been used as a traditional Chinese medicine for approximately 2000 years. Modern pharmacological studies have confirmed that ginseng can exert antifatigue effects through eliminating free radicals, promoting the utilisation of glycogen, reducing the accumulation of lactic acid (LA) and protecting the central nervous system (Xiang et al., 2008). Polysaccharides are another main medicinal component in ginseng. In addition to the reported antitumor, lowering blood sugar, antiradiation and other biological activities (Sun, 2011), ginseng polysaccharide present antifatigue effects (Wang et al., 2010).

Polysaccharides are biological macromolecules that are formed by more than 10 monosaccharides through glycosidic bonding. The biological activities of polysaccharides are based on their structure features. Starch-like neutral ginseng polysaccharides and pectin-like polysaccharides have been reported since 1960s (Ovodov and Solov’eva, 1966). The former, which is composed mainly of α-(1→4)-Glc, has been proven to possess immunostimulating, antitumor and antixidation effects (Ni et al., 2010; Li et al., 2019; Luo et al., 2008). By contrast, ginseng pectin is an acid heteropolysaccharide that is composed of arabinogalactans, rhamnogalacturonan and homogalacturonan domains; it has more obvious advantages in the hypoglycemic and anti-fatigue activities (Jiao et al., 2014). A growing number of studies have shown that the molecular configuration of polysaccharides has a great effect on their biological functions as natural biomacromolecules. Research has confirmed that the antitumor and immunomodulatory effects of some polysaccharides are dependent on their triple helix conformation. When their helix structure is destroyed, their corresponding biological activities decrease or even disappear (Falch et al., 2000). Sulphation and carboxymethylation derivation cause changes in the conformation of polysaccharides by loosening the spherical conformation of polysaccharide molecules, exposing functional groups and increasing the interaction between polysaccharides and receptors (Tao et al., 2009). Therefore, studies on polysaccharides conformation are of great importance to research on structure–activity relationships. Moreover, the conformational characteristics of polysaccharides may be highly dependent on their molecular weights (Wang et al., 2016). However, studies on the solution configuration of ginseng polysaccharides remain scarce so far.

In consideration of the above deficiencies, in this study, polysaccharides with different molecular weights were obtained from steamed ginseng *via* ultrafiltration, and their physicochemical properties, chain configurations and antifatigue activities were evaluated.

## Materials and Methods

### Preparation of Steamed Ginseng Polysaccharides

The fresh *P. ginseng* was obtained from Ji'an, Jilin Province, China, and authenticated by Professor Bo Li (School of Pharmacy, Changchun University of traditional Chinese Medicine). Steamed ginseng was prepared in accordance with a previous method (Jiao et al., 2014). After washing with distilled water, fresh ginseng was steamed in an autoclave at 120°C for 3 h and then air-dried for 10–15 days.

The dried steamed ginseng was extracted under reflux extraction for 2 h with 1 L of 95% ethanol. The residue was washed with distilled and further extracted with hot water three times at 100°C. The resulting extract was combined, concentrated under reduced pressure and then lyophilized. The lyophilized product (10 g) was dissolved in 5 L of distilled, and then the protein was removed *via* the Sevag method (Staub, 1965). Finally, the deproteined product, enriched in the polysaccharide, was dialyzed, concentrated and freeze-dried to yield WSGP (6.06 g).

WSGP was dissolved in distilled water at a concentration of 1%. The solution was fractionated using a hollow fibre UF membrane system (average molecular weight [Mw] cut-off of 30,000 Da). The permeate and retentate were collected separately, concentrated and lyophilised to obtain two polysaccharides with different molecular weights. These polysaccharides were further fractionated with a Sephadex G-100 column (2.6 cm × 100 cm) to yield the polysaccharides WSGP-S3 (Mw < 30,000 Da) and WSGP-G3 (Mw > 30,000 Da).

### Chemical Composition Analysis

The phenol–sulphuric acid method was used to determine the total sugar content in polysaccharides (Dubois et al., 1951). Protein content was determined by Bradford’s method (Bradford, 1976), uronic acid content was measured through m-hydroxybiphenyl method (Blumenkrantz and Asboe-Hansen, 1973). The monosaccharide composition of polysaccharides was determined in accordance with previous methods with some modifications (Honda et al., 1989). Briefly, samples (2 mg) were hydrolyzed with anhydrous methanol solution (0.5 ml) containing 1 M hydrochloric acid under nitrogen atmosphere at 80°C for 16 h. The hydrolysate was further hydrolyzed with 2 M TFA (0.5 ml) for 1 h. Ethanol was used to eliminate excess acid. The resulting product was derived with PMP reagent *via* a previously described method (Song et al., 2019). Excessive PMP reagent was removed through chloroform extraction. The PMP derivatives, which were filtered using a 0.22 μm filter membrane, were subjected to a Dionex™ UniMate™ 3000 HPLC system (Thermo Fisher Scientific, United States) fitted with an Inertsil ODS-3 chromatographic column (4.6 ID × 150 mm, 5 μm, Dikma; Technologies Inc, United States) and a UV detector. The injection volume was 10 μL and the column temperature was 40°C.

### Homogeneity Analysis of Polysaccharides

A total of 10 mg polysaccharides was dissolved in 1 ml of distilled water, filtered with a 0.22 μm membrane and analysed using a Dionex™ UniMate™ 3000 HPLC system (Thermo Fisher Scientific, United States) equipped with an RID-10A detector and TSK-G3000 PWXL columns (7.8 mm × 30.0 cm). The mobile phase was ultrapure water, the flow rate was 0.5 ml/min and the injection volume was 20 μL.

### Size-Exclusion Chromatography–Multiangle Laser Light Scattering

The molecular weight and configuration analysis of ginseng polysaccharides were analysed with a size-exclusion exclusion chromatography (SEC)–multiangle laser light scattering (MALLS) system coupled with Agilent 1,260 Infinity II HPLC system, a MALLS photometer (Wyatt Technology Dawn HEIEOS-II, Wyatt, United States) and an ASTRA workstation (ASTRA7.3.0.11, United States). The column (OHpak SB-803HQ column, 8.0 × 300 mm, Shodex, Japan) was eluted with 0.2 M NaCl solution containing 0.02% NaN_3_ at a flow rate of 0.6 ml/min at 35°C. A refractive index increment (dn/dc) of 0.185 ml/g was applied in this experiment (Yan et al., 2019).

The polysaccharide solution (0.5 mg/ml) was filtered with a 0.22 microfiltration membrane and injected into the liquid chromatography at the injection volume of 100 μL. The LS and RI chromatograms were recorded simultaneously. The average molecular number (Mn), Mw, distribution coefficient (Mw/Mn) and conformations of fractions were analysed using ASTRA software.

### Congo Red Experiment

The interaction of Congo red with polysaccharides was used to evaluate polysaccharide conformational characteristics in accordance with a previously described method (Liu et al., 2020). A total of 2 ml of polysaccharide solution (2 mg/ml) was mixed well with an equal volume of 91 μmol/L Congo red reagent. Then, different concentrations of NaOH (1 ml) were added to the mixture to adjust the NaOH concentration of the solution to 0–0.7 mol/L. At the same time, Congo red solution without polysaccharide was used as the experimental control group. The absorbance at 400–700 nm was recorded. The Congo red curve was drawn by using the maximum absorption wavelength as the ordinate and the concentration of sodium hydroxide as the abscissa.

### Circular Dichroism Spectroscopy Assay

The polysaccharides were dissolved in distilled water (1 mg/ml) and applied on a circular dichroism spectropolarimeter (Chirascan Qcd, Applied Photophysics Ltd., Leatherhead, Surry, United Kingdom) at 25°C. The bandwidth was set as 1.0 nm. Data were recorded over 190–350 nm at the scanning speed of 20 nm/min.

### FT-IR Analysis

The polysaccharide sample was dried thoroughly and then mixed and compressed with KBr into a pellet. FT-IR spectra were recorded at 4,000–400 cm^−1^ on a Bruker (Germany) Vector 33 spectrometer.

### Antifatigue Activities of Polysaccharides

#### Animals and Groups

Male Kunming mice (20 ± 2 g) were purchased from Experimental Animal Center directly under Jilin University (Changchun, China), permission code SCXK 2020–0002. All animals were maintained and used in strict accordance with the PR China legislation on the use and care of laboratory animals and the guidelines issued by Experimental Animal Center of Changchun University of Chinese Medicine and were approved by the university committee for animal experiments.

The mice were housed in cages (10 mice per cage) under the following controlled conditions: room temperature of 20–26°C, relative humidity of 40–70% and a light/dark cycle environment for 12 h. The mice were fed freely with standard mouse chow and water.

The mice were randomly divided into 11 groups of 10 mice per group: the normal group, the negative group and nine polysaccharide groups. The mice in the negative control group were treated with 0.2 ml of physiological saline orally. The mice in the polysaccharide-treated groups received 0.2 ml of different concentrations (25, 50 and 75 mg/kg) of WSGP, WSGP-S3 and WSGP-G3. The mice were administered with polysaccharides or physiological saline continuously for 30 days, and their body weights were recorded daily.

#### Effect of Polysaccharides on Weight-Loaded Swimming Time

After the last administration of the experimental treatment, the mice were rested for 30 min for the weight-loaded swimming test as previously reported (Zhang et al., 2009). Each mouse was loaded by attaching a tin wire (9% of the body weight) to their tails. Each mouse was placed individually in a swimming tank containing 30 ± 1 cm of water at 25 ± 1°C. The water was agitated occasionally to keep the mice swimming, and exhaustive swimming time was recorded when the mice became submerged in water for more than 10s.

#### Analysis of Biochemical Parameters

The mice were removed after swimming and allowed to rest for 90 min. Then, the blood samples were collected, allowed to stand at room temperature and centrifuged at 2000 rpm at 4°C for 10 min to obtain serum. The LA, urea nitrogen (BUN), SOD, CAT, GSH-Px and MDA level contents were determined in according with kit instructions. The liver and hind limb muscles were separated under sterile conditions to determine the content of hepatic glycogen (HG), muscular glycogen (MG).

#### Effect of Polysaccharides on Spleen Proliferation

The spleens and thymuses of the mice mentioned above were separated under sterile conditions. Spleen cells (100 μL) were prepared in accordance with the reported method (Li et al., 2019) and seeded into a 96-well plate at a density of 2 × 106 cells/mL and cultured with or without concanavalin A (ConA, 5 μg/ml) or lipopolysaccharide (LPS, 20 μg/ml) in a 37°C incubator for 68 h. Then, 10 μL of CCK8 reagent was added, and incubation was continued for 4 h. The absorbance at 450 nm was recorded. Three replicate wells were set for each experimental point.

### Data Analysis

Data were displayed as mean ± S.D. The statistical significance of the differences between groups was evaluated by performing ANOVA and Duncan’s-test. Here, *p* < 0.05 was considered as significantly different.

## Result and Discussion

### Characterisation of Polysaccharides

In this study, WSGP was obtained through hot water extraction and firstly purified *via* the Sevag method. The protein content of lower than 2% was detected in WSGP by Bradford’s method. Then WSGP was fractionated *via* ultrafiltration and with Sephadex G-100. Subsequently, the two polysaccharides WSGP-G3 and WSGP-S3 were obtained with the yields of 23.28 and 8.01%, respectively (Table 1). Monosaccharide analysis showed that WSGP-S3 and WSGP-G3 consisted of six monosaccharides: Rha, GlcA, GalA, Glc, Gal, and Ara. Therefore, these polysaccharides were acid heteropolysaccharides. WSGP-S3 contained Gal (32.02%) and Rha (35.48%) as its dominant monosaccharide components, together with small amount of GalA, Glc, Ara, and GlcA (Table 1). We previously obtained the steamed ginseng acid polysaccharide GPS-1 with significant antihyperglycemic activity. The monosaccharide composition of GPS-1 was similar to that of WSGP-S3. The total amount of Gal and Ara in GPS-1 was nearly 70%, and it contained type I arabinogalactans, arabinogalactans-II and rhamnogalacturonan I domains (Jiao et al., 2020) Lee et al. (2019) obtained a similar red ginseng polysaccharide, which showed better immunostimulating activity than other fractions. Ara was the predominant monosaccharide in WSGP-G3, wherein it accounted for nearly 60% of the polysaccharide content. This is the first time that we obtained a ginseng polysaccharide with such a high content of Ara content. This result indicated that the monosaccharide ratios of the two polysaccharides were significant different. This difference might result in difference in their physicochemical properties and biological functions.

**TABLE 1:** Yield and monosaccharide analysis of WSGP-S3 and WSGP-G3.

Group	Yield (%)	Monosaccharide composition (mol%)					
		Rha	GalA	Glc	Gal	Ara	GlcA
WSGP-S3	8.01	35.48	6.98	5.98	32.02	16.95	2.67
WSGP-G3	23.28	9.96	6.49	7.65	16.56	57.97	0.73

### Analysis of the Homogeneities and Molecular Weights of WSGP-S3 and WSGP-G3

**FIGURE 1:**
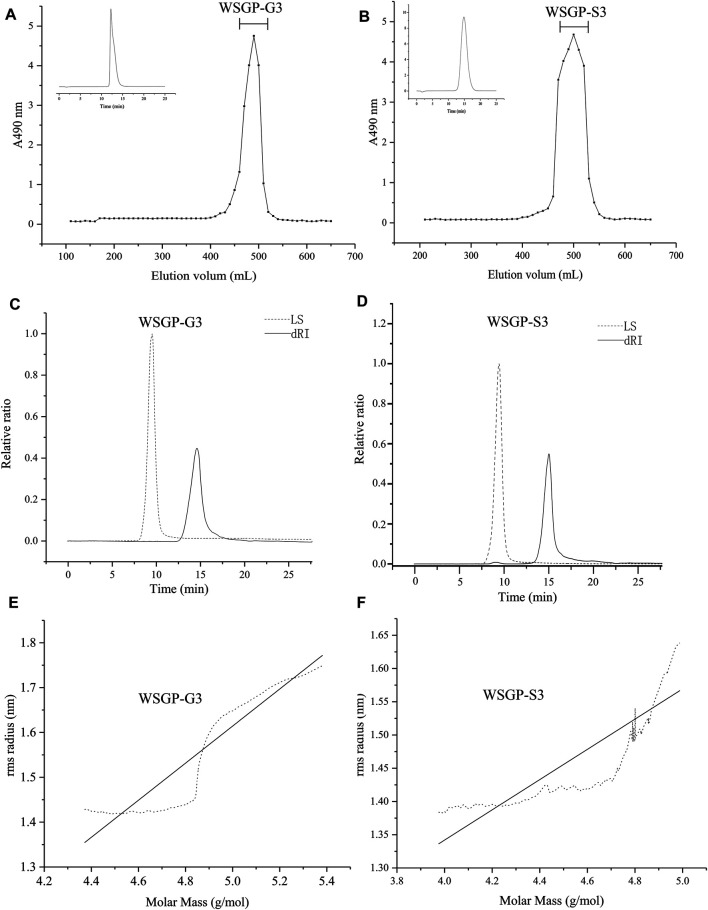
Gel permeation chromatography profiles for: **(A)**WSGP-G3; **(B)** WSGP-S3; SEC-MALLS chromatograms by MALLS and DRI for: **(C)** of WSGP-G3; **(D)** WSGP-S3; **(C)** Plot of log(S2)z^1/2^ vs. the log*M*w for: **(E)** WSGP-G3; **(F)** WSGP-S3.

In this study, homogeneities were firstly analysed using a HPLC system. The HPLC profiles of WSGP-S3 and WSGP-G3 showed a signal narrow symmetric peak, showing that two homogeneous polysaccharides were obtained (Figures 1A,B). The chemical composition, molecular weight and chain conformation of polysaccharides have been studied extensively because of their importance in explaining structure–activity relationships. SEC–MALLS is a highly convenient method for determining the absolute molecular weight and distribution of polymer compounds, and it has high sensitivity for polymers with high molecular weights (Viebke et al., 1995). The polysaccharides were further analysed using a MALLS system coupled with a SEC system. The results are provided in Table 2. Consistent with the HPLC results, SEC-MALLS and SEC-RID results revealed that WSGP-G3 and WSGP-S3 had a single peak (Figures 1C,D). The average molecular weights of the two samples were 4.86 × 10^4^and 2.03 × 10^4^, respectively. The polydispersity (Mw/Mn) values of WSGP-G3 and WSGP-S3 were 1.17 and 1.26, respectively. These values were close to 1, proving that the molecular mass distribution of these polysaccharides was narrow and homogeneous.

**TABLE 2:** SEC-MALLS analysis of polysaccharides of WSGP-S3 and WSGP-G3.

Group	*M*w (Da)	*M*n	*M*w*/M*n	*M*z	Slope
WSGP-S3	2.03 × 10^4^	1.61× 10^4^	1.26	2.76× 10^4^	0.23
WSGP-G3	4.86 × 10^4^	4.13× 10^4^	1.17	5.72× 10^4^	0.41

SEC-MALLS can also provide information about polysaccharide conformation in solution (Bello-Perez et al., 1998). The relationship between the radius of gyration (Rg) and molecular weight can be described using the following formula, which can reflect polysccharide conformation.

$$<S^{2}{>}_{Z}^{1/2}=KM_{W}^{V}$$

Where $<S^{2}{>}_{Z}^{1/2}$ is the Rg, and v is the linear fitting slpoe of Rg-Mw plot that depicts the conformation of the macromolecules (Raoufi et al., 2018). According to the literature, a molecule with a slope of 0.3 is generally considered as spherical, that with a slope between 0.5–0.6, is linear with a Gaussian coil configuration and that with a slope greater than 0.6 has an extended structure (Wang et al., 2009). In this work, the slope of WSGP-S3 was 0.23 (Figure 1F), indicating that in aqueous solution; it existed as a branched cluster with a nearly spherical configuration. WSGP-G3, which had a slope of 0.43 might have an approximately coil configuration in solution (Figure 1E).

### Congo Red Analysis of WSGP-G3 and WSGP-S3

**FIGURE 2:**
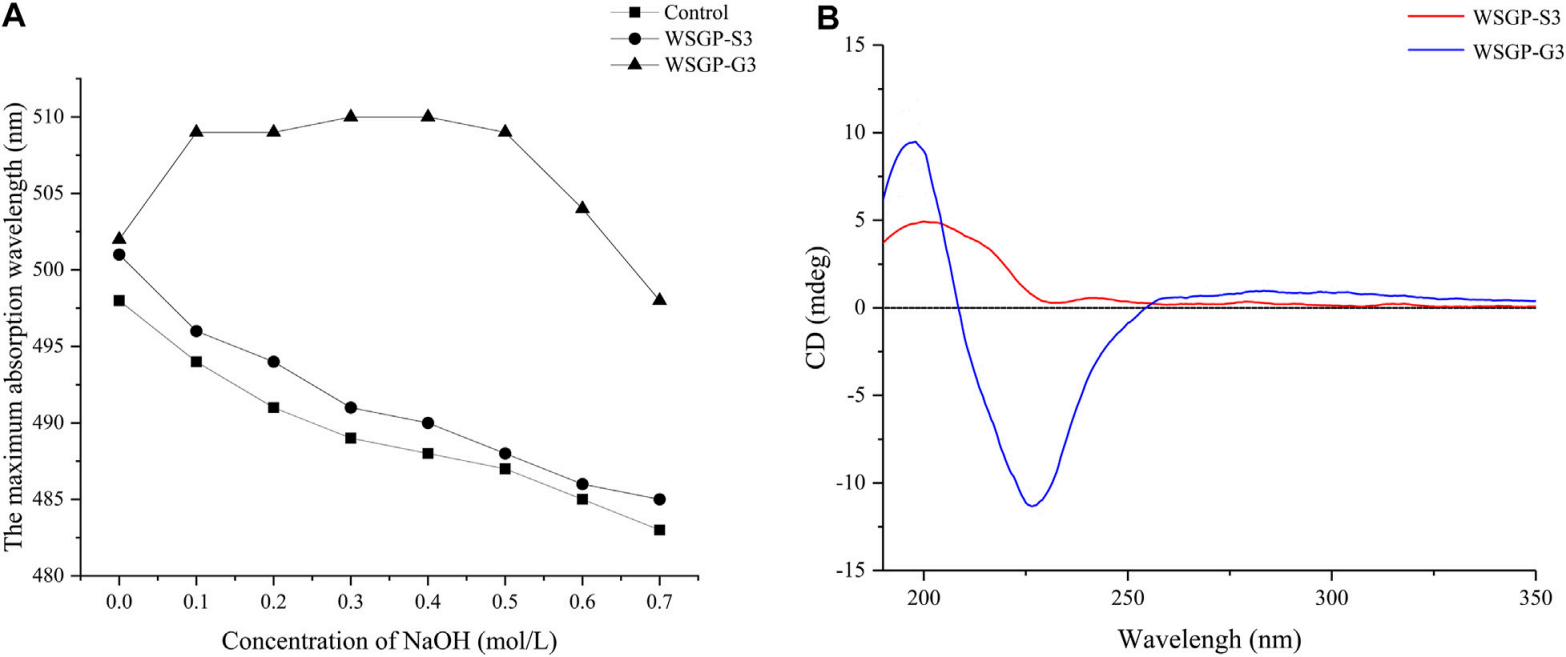
Characteristics of WRPG-G3 and WRPG-S3: **(A)** Changes in absorption maximum of Congo red-WRGP-G3 and red-WRGP-S3 complex at various concentrations of NaOH; **(B)** CD spectra of WRPG-S3 and WRPG-G3.

Congo red is an acid dye with the molecular formula of C_32_H_22_N_6_O_6_S_2_Na_2_. Congo red can form a stable complex with polysaccharides with helical configurations. The maximum absorption wavelength of the polysaccharide–Congo red complex is red-shifted in a certain concentration range of NaOH solution. When the concentration of NaOH is increased, the hydrogen bond between hydroxyl groups in polysaccharides is weakened; this effect leads to the destruction of the helix conformation. Therefore, this property can be used to detect whether the polysaccharide has a helical structure (Wang et al., 2016). As shown in Figure 2A, the complexation of WSGP-G3 with Congo red resulted in a characteristic increase in the maximum absorption wavelength over the a NaOH concentration of 0–0.5 mol/L and indicated that WSGP-G3 presented an ordered, three-stranded spiral structure. When the concentration of NaOH higher than exceeded 0.5 M, the helical conformation of WSGP-G3 was destroyed, and the maximum absorption wavelength decreased rapidly. The maximum absorption wavelength of WSGP-S3 decreased with the increase in NaOH concentration. This behavior indicated that inferred WSGP-S3 did not have a triple helix structure but instead had a random coil conformation.

### Circular Dichroism Spectroscopy Analysis of WSGP-S3 and WSGP-G3

Circular dichroism spectroscopy is an optical method that is used to study the asymmetry of the three-dimensional molecular spatial structures of biological macromolecules (Bystrickx et al., 1995). In this work, circular dichroism was used to understand the chain configuration of WSGP-S3 and WSGP-G3. As shown in Figure 2B, an obvious positive Cotton effect at 197.60 nm and a negative Cotton effect at 224.68 nm were observed in the CD spectrum of WSGP-G3; these effects were caused by the influence of helix-like and sheet-like structures and predominance of the α-anomer (Morris et al., 1975). This result was consistent with the results of Congo red staining. The CD spectrum of WSGP-S3 was different from that of WSGP-G3. The CD spectrum of WSGP-S3 almost lacked an absorption band at approximately 200 nm, indicating that the negative Cotton effect had attenuated or was absent, thus proving that the ordered helical structure of the polysaccharide had been weakened. The experiments once again proved that WSGP-S3 and WSGP-G3 possessed different chain configurations. The negative Cotton effect that appeared between 200–230 nm in the spectra of WSGP-G3 and WSGP-S3 was caused by the n→π* transitions of the carboxyl group; this result was consistent with the result of monosaccharide analysis (Liu et al., 2020).

### FT-IR Analysis

**FIGURE 3:**
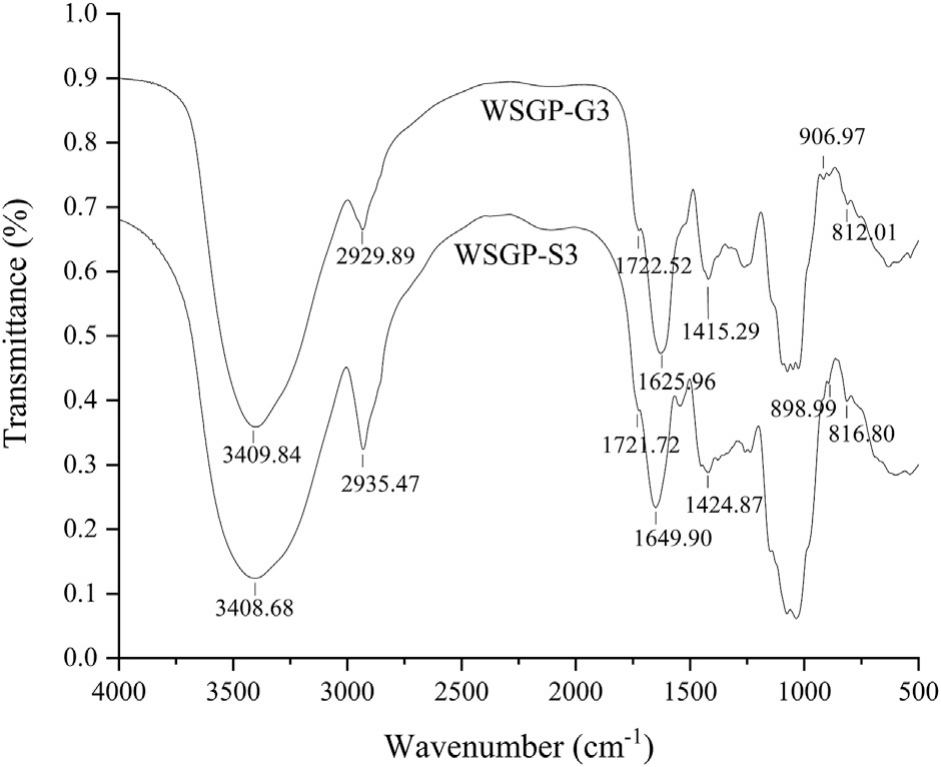
FT-IR spectra of the polysaccharides WSGP-G3 and WSGP-S3.

FT-IR analysis was performed to determine the characteristic absorptions of WSGP-G3 and WSGP-S3. As shown in Figure 3, the FT-IR spectra of WSGP-G3 and WSGP-S3 had strong absorption bands at 3,409.84 and 3,408.68 cm^−1^ that were assigned to O–H stretching vibration. The signals at approximately 2,930 cm^−1^ were attributed to C–H stretching vibration. The absorption peaks at 1,625.96–1,635.9.90 and 1,415.29–1,424.87 cm^−1^ were caused by the asymmetric and symmetric stretching vibrations of the carboxyl groups in the two polysaccharides (Li et al., 2020).The peak observed at 1722.52 (WSGP-G3) and 1721.72 cm^−1^ (WSGP-S3) were ascribed to the C=O band of the carboxyl groups (Gan et al., 2010). The FT-IR spectra exhibited absorption peaks at 812.01–816.80 cm^−1^ and 898.99–906.97 cm^−1^. The presence of these peaks indicated the existence of α- and β-configuration glycosidic bonds in WSGP-G3 and WSGP-S3.

### Antifatigue Activity of Polysaccharides

#### Effect of Polysaccharides on Weight and Organ Indices

**FIGURE 4:**
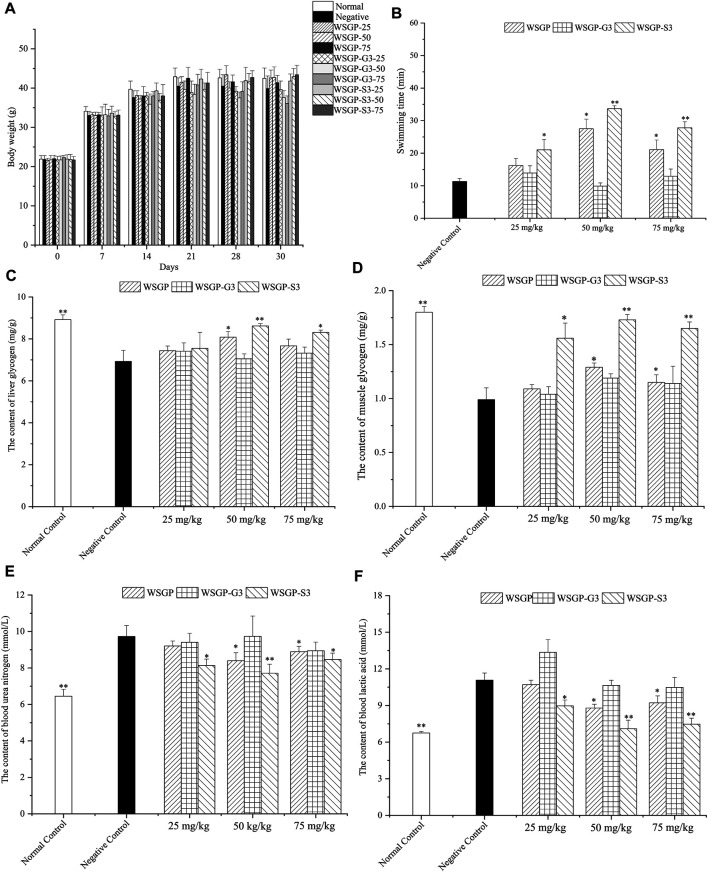
Effect of the polysaccharides on the fatigued mice. **(A)** The changes of body weight; **(B)** Swimming time; **(C)** Liver glycogen; **(D)** Muscle glycogen; **(E)** Blood urea nitrogen; **(F)** Blood Lactic acid. ^*^ *p* < 0.05, ^**^,*p < 0.01 vs. the negative control group.

The effect of steamed ginseng polysaccharides on the body weight, thymus index and spleen index of the mice is shown in Figure 4A and Table 3. As the period of administration with steamed ginseng polysaccharides was prolonged, the weight of the mice increased gradually. However, the weekly increase in the weight of the mice in each group was not significant (*p*＞0.05).

**TABLE 3:** Effects of the polysaccharides on ConA- or LPS-induced lymphocyte proliferation in mouse *in vivo*.

Group	Thymus index (mg/g)	Spleen index (mg/g)	Lymphocyte	
			ConA	LPS
Normal control	3.35 ± 0.18	7.95 ± 0.67	0.76 ± 0.013	0.66 ± 0.023
Negative control	2.33 ± 0.38^a^	5.34 ± 0.68^a^	0.43 ± 0.009^b^	0.31 ± 0.022
WSGP-25	2.38 ± 0.48	5.78 ± 0.66^b^	0.60 ± 0.007	0.48 ± 0.013
WSGP-50	3.25 ± 0.29^c^	6.52 ± 0.48^c^	0.76 ± 0.022^c^	0.61 ± 0.006^d^
WSGP-75	3.19 ± 0.49^c^	6.34 ± 0.36	0.72 ± 0.010^b^	0.58 ± 0.003
WSGP-G3-25	2.69 ± 0.12	5.40 ± 0.18	0.42 ± 0.046	0.52 ± 0.025
WSGP-G3-50	2.35 ± 0.42	5.58 ± 0.10	0.50 ± 0.102	0.51 ± 0.014
WSGP-G3-75	2.89 ± 0.59	5.50 ± 0.58	0.51 ± 0.021	0.54 ± 0.008
WSGP -S3-25	3.11 ± 0.12^c^	6.62 ± 1.08	0.75 ± 0.010	0.62 ± 0.004
WSGP -S3-50	3.93 ± 0.24^d^	9.66 ± 2.30^d^	0.99 ± 0.045^d^	0.79 ± 0.022^d^
WSGP -S3-75	3.49 ± 0.18^c^	8.58 ± 1.94^c^	0.91 ± 0.023^d^	0.73 ± 0.016^d^

Values were expressed as mean ± SD (n = 10).

a *p* < 0.05 vs. normal control group.

b *p* < 0.05 vs. normal control group.

c *p* < 0.01 vs. negative control group.

d *p* < 0.05 vs. negative control group.

Thymus and spleen are important immune organs, and their organ index can reflect the immune function of the body to a certain extent (Ni et al., 2010). The results of this work also showed that the thymus and spleen index in the WSGP and WSGP-S3 treated groups had significantly increased compared with those in the control group (*p* < 0.05 or *p* < 0.01), and the optimal effect was observed under treatment with 50 mg/kg polysaccharides. However, WSGP-G3 had no significant effect on the increase in the organ index within the tested dose range.

#### Effect of Polysaccharides in the Weight-Loaded Swimming Test

The improvement in body endurance is the most direct manifestation of delayed physical fatigue. The antifatigue capability of natural products is usually evaluated using a load-bearing swimming animal model (Bogdanova et al., 2013). Therefore, in this study, the effect of steamed ginseng polysaccharides on exhaustive swimming time was firstly investigated. As illustrated in Figure 4B, compared with the control group, treatment with 25–75 mg/kg WSGP and WSGP-S3 significantly prolonged the exhaustive swimming time of mice under weight-bearing conditions (*p* < 0.05 or *p* < 0.01). Meanwhile, treatment with 50 mg/kg WSGP and WSGP-S3 showed the best effect on prolonging the exhaustive time of mice. Treatment with 25 or 75 mg/kg WSGP-G3 prolonged exhaustive time; this effect, however, was not significantly different from the effect of the control treatment (*p* > 0.05). These results demonstrated that WSGP and WSGP-S3 had a significant influence on prolonging the exhaustive swimming time of fatigued mice, and at the same concentration, WSGP-S3 was more effective than WSGP.

#### Effect of the Polysaccharides on HG and MG Content

The occurrence of exercise-induced fatigue is closely related to factors, such as energy consumption, metabolite accumulation and internal environmental changes. During long-term exercise, energy is mainly supplied through the combination of glycolysis and aerobic oxidation and is firstly supplied by the decomposition of MG. With the continuous extension of exercise time, MG is consumed in large quantities, blood sugar is reduced and the body manifests fatigue symptoms; energy is supplied by LG when MG is consumed (Huang et al., 2015). In other words, the content of LG and MG plays a crucial role in maintaining blood sugar levels, and LG and MG reserves can improve fatigue. As shown in Figures 4C,D, compared with those in the control group, the levels of LG and MG in the negative control mice decreased significantly (*p* < 0.05 or *p* < 0.01). By contrast, the LG levels of the 25, 50 and 75 mg/kg WSGP-treated groups increased by 10.24, 16.59 and 10.68%, respectively. The LG levels of the 25, 50 and 75 mg/kg WSGP-S3-treated group increased by 17.60, 24.39 and 19.91%, respectively. Although WSGP-G3 administration could increase LG levels in fatigued mice, the increase in LG content was less than 10% within the tested dosages, and no similar results were observed in the investigation of MG levels. WSGP-S3 significantly recovered the MG levels of fatigued mice. Compared with that in the control group, the MG level in the 50 mg/kg WSGP-S3-treated group had increased by 74.75%. These results suggested that administration with WSGP and WSGP-S3 could significantly increase LG and MG reserves. At the same concentration, WSGP-S3 showed a stronger effect than WSGP, its optimal dosage was 50 mg/kg. These results indicated that WSGP-S3 played a major role in the antifatigue effect of WSGP.

#### Effect of Polysaccharides on BUN and LA Contents

Prolonged exercise can cause the accumulation of various fatigue-related metabolites (Li et al., 2016). During strenuous exercise, the inadequate oxygen supply to the body accelerates glycolysis and produces a large amount of LA. At the same time, the capability of body to catabolise protein is strengthened during strenuous exercise, resulting in an increase in BUN level, which is positively correlated with exercise intensity. Therefore, reducing the LA and BUN production or accelerating LA and BUN elimination can improve fatigue or accelerate its elimination. As shown in Figures 4E,F, WSGP and WSGP-S3 treatment significantly reduced BUN and LA levels in mice compared with the control treatment (*p* < 0.05 or *p* < 0.01). In accordance with the above results, 50 mg/kg WSGP-S3 showed the strongest antifatigue effect. Treatment with 50 mg/kg WSGP-S3 decreased LA and BUN contents by 35.95 and 20.76%, respectively. Treatment with 50 mg/kg WSGP decreased LA and BUN contents in WSGP groups by 20.60 and 13.67%, respectively. No significant changes were observed in LA and BUN levels in WSGP-G3 treated mice. The results of the exhaustive swimming test and the biochemical parameters indicated that the effect of WSGP-S3 on fatigue resistance was more significant than that of WSGP-G3.

The biological activity of polysaccharides is closely related to their primary structure, which is affected by backbone properties (molecular weight, monosaccharide composition, sugar residues sequences, linkage type and anomeric carbon configuration) and branch properties (branched or unbranched and branch type, position and length). Amongst these properties, molecular weight is the key factor that affects the biological activities, such as immunity (Sun et al., 2012), antioxidant (Xu et al., 2016) and antitumor activities (Kojima et al., 1986), of polysaccharides. Furthermore, this relationship is not linear. Excessive high or low molecular weights are not conducive to the development of exertion of the activities of polysaccharides (Bhadja et al., 2016). The present work found that although the molecular weight of WSGP-G3 was 2.4 times higher than that of WSGP-S3, the antifatigue effect of WSGP-S3 was markedly higher than that of WSGP-G3. This result indicated that the antifatigue activity of the ginseng polysaccharides might be negatively correlated with their molecular weights. Through continuous in-depth research on polysaccharides, researchers have found that the biological functions of polysaccharides are also closely related to their conformational features, which are closely dependent on molecular weights, in addition to their primary structure (Wu et al., 2013). The above results indicated that WSGP-S3 and WSGP-G3 exhibited different conformations in aqueous solutions. However, further research is required on the relation-ship between antifatigue effecs and the solution conformatios of the polysaccharides(l.421–422).

### Effect of Polysaccharides on the Antioxidant System

**FIGURE 5:**
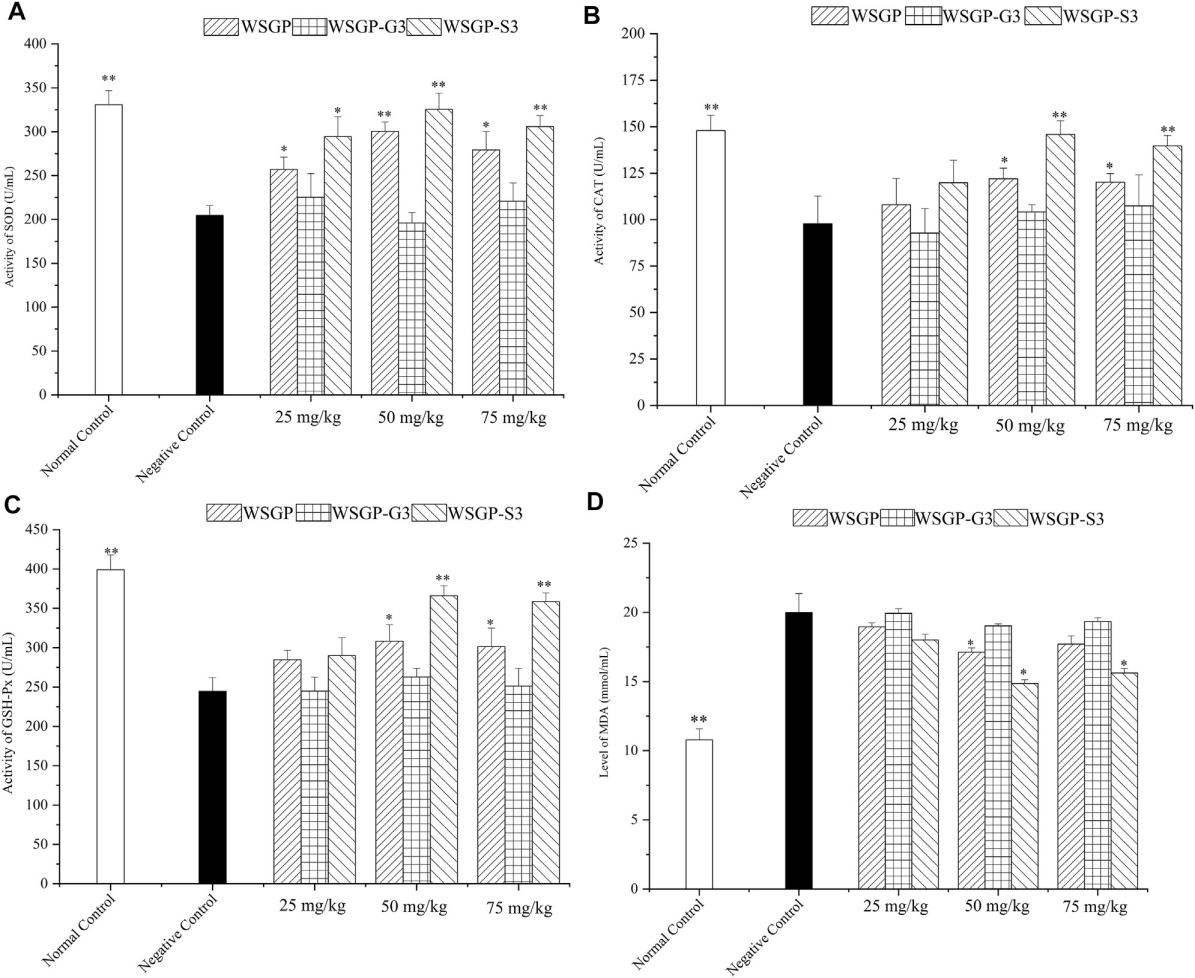
Effects of the polysaccharides on the antioxidant system of fatigued mice. **(A)** Activity of SOD; **(B)** Activity of CAT; **(C)** Activity of GSH-Px; **(D)** Level of MDA. ^*^ *p* < 0.05, ^**^ *p* < 0.01 vs. the negative control group.

Intense exercise can cause the production of excessive ROS and free radicals, which can damage organelles, such as mitochondria and cell membranes, and decrease the activity of antioxidant enzymes, such as SOD (Wu et al., 2013). When the redox equilibrium is disrupted, tissue integrity is affected, and cell membrane structure and cell function are damaged, thus affecting the normal function and energy metabolism of the body. Therefore, oxidative stress plays an important role in the development of fatigue and oxidative stress and has good application prospects in fatigue treatment (Chi et al., 2015). Recently, a large number of studies have confirmed that plant polysaccharides can play an antifatigue role by improving oxidative stress systems in exercise-induced fatigue (Chi et al., 2015). The results of the present work showed that compared with those under the control treatment, the activities of SOD (Figure 5A), CAT (Figure 5B) and GSH-Px (Figure 5C) significantly increased (*p* < 0.05 or *p* < 0.01) under the continuous administration of 50–75 mg/kg WSGP or WSGP-S3 for 30 days. Meanwhile, MDA levels in WSGP or WSGP-S3-treated group obviously decreased. As shown by previous results, the optimal dose of WSGP or WSGP-S3 was 50 mg/kg, and WSGP-S3 had stronger antioxidant activity than WSGP at the same concentration. By contrast, the enzyme activities and MDA level in the WSGP-G3 group were not obviously different from those in the control group (Figure 5D). These results indicated that WSGP and WSGP-S3 could improve the antioxidant system of fatigued mice. This result was consistent with previously reported results. Many studies have demonstrated that ginseng polysaccharides possess antioxidant activities (Luo and Fang, 2008; Jiao et al., 2014). We also preciously found that acid polysaccharides from steamed ginseng could improve alloxan-induced oxidative damage (Jiao et al., 2014). The balance of free radicals is crucial for maintaining health and relieving fatigue. Excessive free radicals in the body, if not cleared in time, attack macromolecules and cellular organs and induce lipid peroxidation, thus damaging the myocardium and skeletal muscle (Valko et al., 2007). In this experiment, the MDA content in the control mice increased. This effect confirmed that fatigue was related to oxidative damage. A variety of plant polysaccharides improve antioxidant capacity by providing energy, promoting metabolism and removing excessive free radicals, thus playing an antifatigue role (Chi et al., 2015). In the present study, in the WSGP groups and WSGP -S3 groups, the SOD, CAT and GSH-PX activities in liver tissue increased significantly and MDA level decreased. These effect indicated that WSGP and WSGP-S3 had an important role in maintaining antioxidant enzyme activity and alleviating lipid peroxidation. These results showed that the polysaccharides could exert an antifatigue effect by repairing oxidative damage. Furthermore, uronic acid is an important index that reflects the antioxidant activity of polysaccharides, that is, acidic polysaccharides are usually potent antioxidants (Al-Sheraji et al., 2012; Wu et al., 2013). Wang et al. (2010) also found that acid ginseng polysaccharide possess stronger antifatigue effects than neutral ginseng polysaccharide. However, in this work, WSGP-S3 and WSGP-G3 contained almost equal amount of uronic acid but exhibited significant differences in antioxidant activity. Therefore, the differences in physical and chemical properties and solution conformations of steamed ginseng polysaccharides might have great contributious to their antioxidant effects.

### Effect of Steamed Ginseng Polysaccharides on Lymphocyte Proliferation

Next, the effect of the polysaccharides on lymphocyte proliferation was investigated. Compared with the control group, WSGP-S3 (50 and 75 mg/kg) effectively promote dlymphocyte proliferation (Table 3). It could also promote ConA or LPST-induced mouse T- or B-lymphocyte proliferation (*p* < 0.01). By contrast, lymphocyte proliferation in the WSGP-G3 treated group was obviously affected relative to that in the control group. Immunoenhancement activity is one of the essential bioactivities associated with ginseng polysaccharides (Ni et al., 2010). The continuous administration of WSGP-S3 improved the immune capability of mice, and the improvement in immunity enhanced the capability of the mice to resist fatigue. Hence, WSGP-S3 had a significant antifatigue effect that might be related to the regulation of metabolism, the restoration of the antioxidant system and the enhancement of the immune system in fatigued mice.

## Conclusion

Two acid polysaccharides (WSGP-S3 and WSGP-G3) with different molecular weights were obtained from steamed ginseng. WSGP-S3 had a lower molecular weight than WSGP-G3. It mainly contained Rha and Gal and might exist in a random configuration in solution. By contrast, WSGP-G3 possessed higher molecular weight than WSGP-S3 and contained Ara as its major sugar. It existed in an ordered solution configuration. WSGP-S3 showed stronger antifatigue activity than WSGP. It exerted its antifatigue activity through the regulation of metabolism and the restoration of the antioxidant system and immune systems. Therefore, WSGP-S3 might have a major contribution to the antifatigue effects of steamed ginseng polysaccharides.

## Data Availability

The raw data supporting the conclusions of this article will be made available by the authors, without undue reservation.
